# Brain Network Adaptability across Task States

**DOI:** 10.1371/journal.pcbi.1004029

**Published:** 2015-01-08

**Authors:** Elizabeth N. Davison, Kimberly J. Schlesinger, Danielle S. Bassett, Mary-Ellen Lynall, Michael B. Miller, Scott T. Grafton, Jean M. Carlson

**Affiliations:** 1 Department of Mechanical & Aerospace Engineering, Princeton University, Princeton, New Jersey, United States of America; 2 Department of Physics, University of California, Santa Barbara, Santa Barbara, California, United States of America; 3 Department of Bioengineering, University of Pennsylvania, Philadelphia, Pennsylvania, United States of America; 4 Department of Electrical and Systems Engineering, University of Pennsylvania, Philadelphia, Pennsylvania, United States of America; 5 University of Oxford Medical Sciences Division, John Radcliffe Hospital, Headington, Oxford, United Kingdom; 6 Behavioural and Clinical Neuroscience Institute and Department of Psychiatry, University of Cambridge, Cambridge, United Kingdom; 7 Department of Psychological and Brain Sciences, University of California, Santa Barbara, Santa Barbara, California, United States of America; Hamburg University, Germany

## Abstract

Activity in the human brain moves between diverse functional states to meet the demands of our dynamic environment, but fundamental principles guiding these transitions remain poorly understood. Here, we capitalize on recent advances in network science to analyze patterns of functional interactions between brain regions. We use dynamic network representations to probe the landscape of brain reconfigurations that accompany task performance both within and between four cognitive states: a task-free resting state, an attention-demanding state, and two memory-demanding states. Using the formalism of hypergraphs, we identify the presence of groups of functional interactions that fluctuate coherently in strength over time both within (task-specific) and across (task-general) brain states. In contrast to prior emphases on the complexity of many dyadic (region-to-region) relationships, these results demonstrate that brain adaptability can be described by common processes that drive the dynamic integration of cognitive systems. Moreover, our results establish the hypergraph as an effective measure for understanding functional brain dynamics, which may also prove useful in examining cross-task, cross-age, and cross-cohort functional change.

## Author Summary

The human brain is a complex system in which the interactions of billions of neurons give rise to a fascinating range of behaviors. In response to its changing environment—for example, across situations involving rest, memory, focused attention, or learning—the brain dynamically switches between distinct patterns of activation. Despite the wealth of neuroimaging data available, the immense complexity of the brain makes the identification of fundamental principles guiding this task-based organization of neural activity a distinct challenge. We apply new techniques from dynamic network theory to describe the functional interactions between brain regions as an evolving network, allowing us to understand these time-dependent interactions in terms of organizing characteristics of the whole network. We examine patterns of neural activity during rest, an attention-demanding task, and two memory-demanding tasks. Using network science techniques, we identify groups of brain region interactions that change cohesively together over time, both across tasks and within individual tasks. By developing tools to analyze the size and spatial distributions of these groups, we quantify significant differences between brain network dynamics in different tasks. These tools provide a promising method for investigating how the changing brain network properties of individuals correspond to task performance.

## Introduction

An essential characteristic of the human brain is the ability to transition between functional states in synchrony with changing demand. A central focus in neuroscience involves quantifying this adaptability and understanding the underlying brain organization that supports it. Several studies have accomplished this with functional MRI techniques by delineating changes in regional blood-oxygen-level-dependent (BOLD) signal associated with different cognitive tasks, or between task states and task-free (resting [1], [2]) states [3], [4]. However, this approach, which examines the magnitude of brain activity alone, is unable to completely describe the complex correlation structure linking spatially segregated neural circuits. In particular, while providing crucial insight into the spatial structure and anatomical distribution of functional activity and how it differs between task and resting states, these methods are not well suited to probe the intrinsic organization of the dynamics of task-driven transitions between cognitive states, or co-evolving associations among brain regions throughout a particular task.

Recent advances in network science provide tools to represent and characterize the functional interactions between brain regions forming cognitive systems. In this formalism, brain regions are represented as network nodes and functional connections (estimated by statistical similarities between BOLD signals [5]) are represented as network edges [6], [7]. These approaches enable the statistically principled examination of large-scale neural circuits underlying cognitive processes, and have enabled quantitative comparisons *between* circuits [8], [9]. Indeed, a growing literature provides evidence that individual tasks may elicit specific functional connectome configurations [10], while maintaining a relatively stable functional backbone reminscent of the connectome configuration evident in the resting state [11].

Nevertheless, these studies have focused on examining task or cognitive states as separate and independent entities, and tools to quantify how brain networks reconfigure between these task states remain significantly underdeveloped. Initial efforts to examine reconfiguration properties of brain networks have focused on quantifying properties of dynamic functional connectivity at rest [12]. A relatively few studies have begun to examine reconfiguration properties during task states [13]–[17] or across a series of brain states accompanying behavioral change [18]–[21]. These studies have robustly demonstrated that functional connectome patterns change during task execution, and that individual differences in these reconfiguration properties have implications for task performance [13], [18]–[20].

In this paper, we ask a complementary set of questions that focus on sets of functional connections rather than on the entire functional connectome pattern. We ask whether sets of functional connections evolve independently within or across brain states, or whether they evolve cohesively, each set controlled by a common regulatory driver. To answer this question, we employ recently developed dynamic network science methods to estimate brain functional networks in one-minute time intervals as 86 participants engage in four task states: a task-free resting state, an attention-demanding state, and two memory-demanding states. We treat the evolving patterns of functional connectivity as temporal, or dynamic, networks [14], [15], [18], [19], [21], [22] and estimate the pairwise correlation between the strengths of functional interactions over time in order to identify groups of functional interactions which display similar changes in strength within and across task states. These groups of network edges with similar dynamic patterns, known as *hyperedges*, have been used to quantify the co-evolution in functional brain networks over the course of a learning task [23]. Our goal is to adapt this dynamic network science method to investigate the organization of evolving functional correlations both within and between task-specific cognitive states, using hyperedges as a measure of co-evolution. We hypothesize that overall, functional interactions between brain regions especially important for particular tasks are likely to be grouped in hyperedges with interactions between regions used strongly in other tasks, capturing co-evolution between task-specific functional networks as they turn off or on together when switching tasks. Furthermore, we expect that those functional correlations that link sets of brain regions whose coordination is crucial to a particular task will be more likely to co-evolve significantly during that task alone.

In this paper, we demonstrate the existence of hyperedges driven by significant co-evolution within groups of functional interactions, both within and across task states. We develop novel network diagnostics to characterize hyperedges according to their structure, anatomy, and task-specificity. These analyses provide a unique window into the adaptability of the brain as it transitions between states and offer quantitative statistics for the comparison of such adaptability across subject cohorts.

## Methods

### Ethics Statement

Informed written consent was obtained from each subject prior to experimental sessions. All procedures were approved by the University of California, Santa Barbara Human Subjects Committee.

### Tasks

Subjects engaged in a resting-state (task-free) period, as well as three separate tasks designed to engage different cognitive skills and task-specific brain networks: two separate functional runs of the same attention-demanding task, a memory task with lexical stimuli, and a memory task with face stimuli.

**Figure 1:**
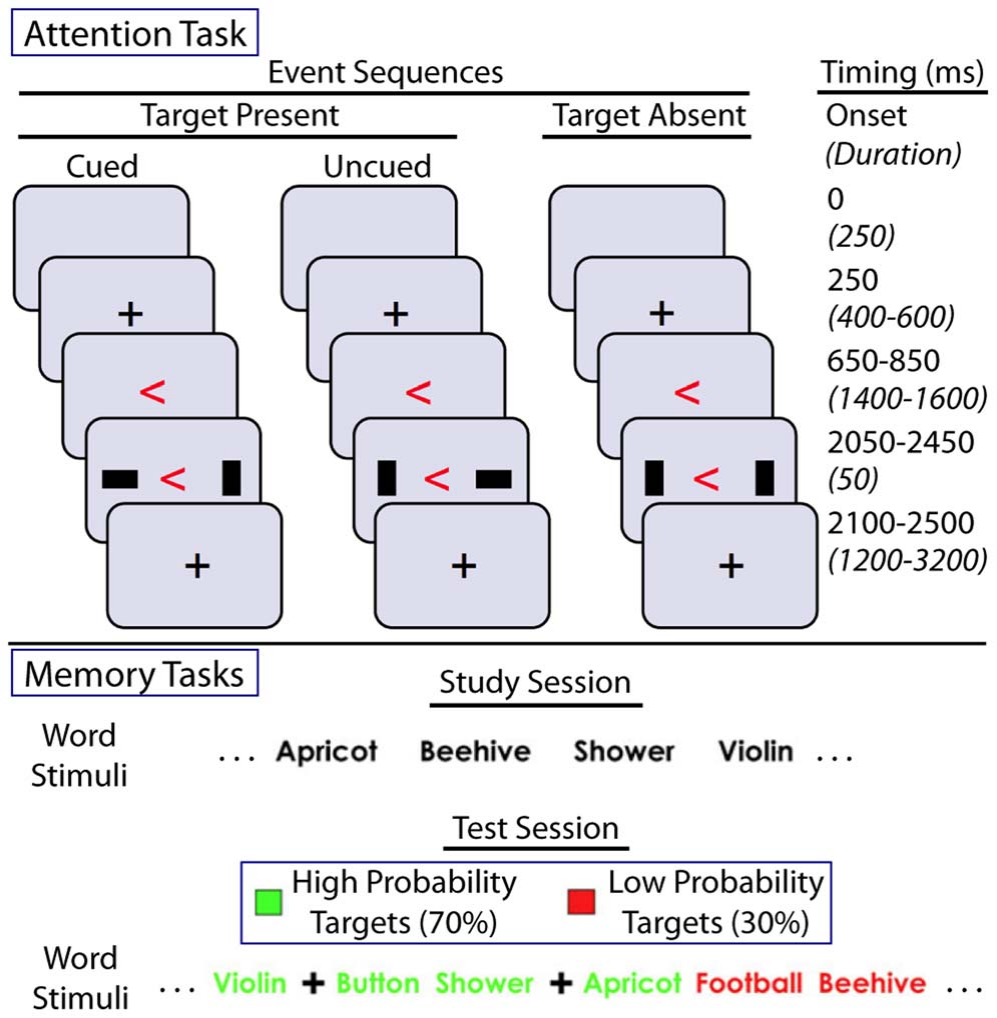
Task setup. **Top panel:** Setup of a single trial sequence in the attention-demanding task. Here, the target stimulus is a horizontal rectangle on either side of the center cross. In each trial sequence, the cross is presented, followed by a cue (arrow) giving probabilistic information about whether and where the target stimulus wil appear, and finally by the stimuli, displayed for approximately 50 ms. The target will either appear as cued, appear in the uncued location, or not appear at all; subjects are required to choose which of these possibilities occurred. **Bottom panel:** Setup of the memory-demanding tasks (same format for word and face memory). In the study session, subjects are presented with a sequence of stimuli. During the test session, another sequence of stimuli is presented; subjects are required to distinguish whether each test stimulus is novel or identical to a stimulus from the study session. Colors of lexical stimuli and colored borders of face stimuli (not pictured) indicate the probability that the test stimulus has been seen before.

During the resting-state period, participants were asked to lie still with their eyes open and look at a blank screen. The attention task (Fig. 1) required subjects to view sequences of visual stimuli on a screen, with the goal of detecting the presence or absence of a target stimulus in each of several test displays. Before each test display, subjects were presented with a cue arrow whose color and direction provided probabilistic information on whether and where the target stimulus might appear. The test display was then flashed for approximately 50 ms, after which the subjects were required to choose whether or not the target stimulus had appeared. In both memory tasks (Fig. 1), 180 previously studied stimuli and 180 novel stimuli were presented to the subjects, who were asked to determine whether each stimulus was “old” or “new” – i.e., whether it had been previously studied. As in the attention task, the memory tasks included probabilistic cues: each stimulus was shown either in a particular color (lexical stimuli) or bordered by a color (face stimuli) which provided subjects with the probability that the stimulus was novel. Face stimuli were drawn from a variety of online faces databases [24]–[29]. For additional experimental details, see [30], [31], and supplemental information therein.

### Imaging

MRI data was acquired at the UCSB Brain Imaging Center from 116 healthy adult participants using a phased array 3T Siemens TIM Trio with a 12 channel head coil. Functional MRI data was taken while each participant engaged in the four tasks described above. This analysis combines two separate functional runs of the same attention task [30]. The sampling period (TR) was 2 s for the rest and attention tasks and 2.5 s for both memory tasks. In addition to functional data, a three dimensional high-resolution T1-weighted structural image of the whole brain was obtained for each participant.

### Image Analysis

#### Structural MRI acquisition and pre-processing

Structural scans were intensity-corrected, skull-stripped, normalized, segmented and parcellated (as described below) using Freesurfer v.5.0.0 cortical reconstruction all with default settings, accessed via the Connectome Mapping Toolkit v.1.2.0 [32]. The starting atlas was the updated Lausanne2008 multi-scale atlas [33]. For each subject, parcellations containing 83, 129, 234, 463 and 1015 regions were generated, covering cortical grey-matter regions, the thalamus, caudate, putamen, pallidum, accumbens area, hippocampus, amygdala and brainstem. The highest-resolution parcellation of 1015 regions was not investigated further, since a large number of regions contained very few or no voxels when the atlas was downsampled into fMRI space.

#### Functional MRI pre-processing and time series analysis

Preprocessing was performed using FSL v5.0 [34]–[36], AFNI v. 2011 12 21 1014 http://afni.nimh.nih.gov [37] and Matlab (2013, The Mathworks, Natick, MA). Functional MRI scans were preprocessed as follows. FSL programs MCFLIRT [38] and fsl motion outliers were used to correct for head motion and derive a volume-by-volume measure of head motion: framewise displacement. Framewise displacement (FD) is calculated as the sum (in mm) of rotational and translational displacements from volume N to N+1 [39]. Next, we performed slice timing correction (AFNI 3dTshift), auto-masked to obtain a brain-only fMRI image (AFNI 3dAutomask), and smoothed the time series at each voxel (AFNI 3dDespike with default parameter settings). Despiking has been shown to reduce the motion-related distance dependent bias in correlation estimates [40]. Each voxel's time series was then detrended with respect to framewise displacement using AFNI 3dDetrend. This uses linear regression to remove variability related to the nuisance regressor, framewise displacement, at each voxel. Runs were only included in the analysis if mean framewise displacement for the run was less than 0.25 mm per frame; this led to 73 fMRI runs (of 763 total runs) being excluded from this analysis. Registration proceeded as follows: a participant's time-averaged fMRI image was aligned to their structural T1 scan using FSL FLIRT boundary-based registration [38], [41], and the inverse of this transformation was applied to all subjects parcellation scales (generated in structural space). Parcellations were downsampled into EPI (AFNI 3dfractionize, voxel centroid voting, requiring 60% overlap), and the mean signal across all the voxels within a given brain region was calculated to produce a single representative time series. The data was not spatially smoothed at any stage.

#### Creation of a hybrid atlas

We sought to create an atlas with low inter-individual and cross-brain variability in the amount of fMRI data acquired per region. Many existing atlases use parcellations that have roughly equal region sizes as measured on structural MRI scans [42]. However, downsampling the atlas from structural MRI voxels to fMRI voxels, along with inhomogeneous fMRI signal-loss, mean that this does not produce equally sized regions in functional MRI space. To mitigate this, we generated a ‘hybrid’ atlas by choosing those regions from various scales of the Lausanne2008 atlas that minimized cross-brain and intra-subject variability in region size. The intra-subject size variability was quantified by the coefficient of variation, defined for each region 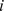 as 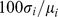, where 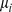 is the mean size of region 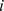 over all subjects and 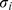 is the standard deviation. Starting with the scale 234 atlas, an iterative process was used to decrease intra- and intersubject variability in region size. Where a region had very few voxels (mean size 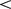 25th percentile), or high variability in size across subjects (coefficient of variation 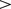 30%), it was tentatively exchanged for a region from the next highest resolution atlas, effectively combining the initial region with other higher-resolution regions subsumed under the same anatomical heading. If this combination of regions decreased the inter-subject or within-subject variability in region size, the combined region was retained. If not, the initial poor quality region was rejected from the “hybrid atlas”. This was repeated until no further combinations of regions could decrease intra- and inter-subject variability while retaining neuroanatomically sensible groupings. Regions were excluded from the analysis altogether if there were fMRI runs in which no data was acquired in that region (frontal pole, entorhinal cortex and temporal pole), or if the inter-subject coefficient of variation was greater than 30% (this applied to 7 of the 8 inferior temporal regions; 1 of the 8 middle temporal regions; 2 of 8 fusiform regions; 1 of the 6 caudal middle frontal regions, and 1 of the 14 precentral regions). Table 1 lists the 194 regions identified by this hybrid atlas. This approach considerably reduced intra-subject variability in region size as well as reducing the inter-subject variability at problematic outlier regions, while minimizing the amount of data that had to be excluded from analysis.

**Table 1:** Brain regions.

Region Name	L	R
lateralorbitofrontal	2	2
parsorbitalis	1	1
medialorbitofrontal	1	1
parstriangularis	1	1
parsopercularis	2	2
rostralmiddlefrontal	5	6
superiorfrontal	9	8
caudalmiddlefrontal	3	2
precentral	7	6
paracentral	1	1
rostralanteriorcingulate	1	1
caudalanteriorcingulate	0	1
posteriorcingulate	2	2
isthmuscingulate	1	1
postcentral	7	5
supramarginal	5	4
superiorparietal	7	7
inferiorparietal	5	6
precuneus	5	5
pericalcarine	1	1
lateraloccipital	5	5
lingual	2	3
fusiform	3	3
parahippocampal	1	1
inferiortemporal	1	0
middletemporal	3	4
bankssts	1	1
superiortemporal	5	5
transversetemporal	1	1
insula	2	2
thalamusproper	1	1
caudate	1	1
putamen	1	1
pallidum	1	1
accumbensarea	1	1
hippocampus	1	1
amygdala	1	1

Anatomical locations of the 194 brain regions used as network nodes in the hyperedge analysis, including the number of regions in left and right hemispheres in each brain area.

### Functional Connectivity

Specific frequencies of oscillations in the BOLD signal have been associated with different cognitive functions. We focus our investigation on low frequency (0.06–0.125 Hz) oscillations in the BOLD signal that have proven useful for examining resting [43], [44] and task-based functional connectivity [18]. The task-related oscillations are posited to be specific to this frequency range, possibly due to a bandpass-filter-like effect from the hemodynamic response function [45]. We apply a Butterworth bandpass filter to isolate frequencies in the (0.06–0.125 Hz) range [46].

To construct a functional brain network, we use the 194 region hybrid atlas, where each region contains a roughly equal number of voxels. These 194 regions represent the network nodes. The 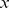, 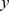, and 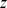 positions of each node are given by the centroid of the voxels which comprise the node. Edge weights in the functional brain network are computed by taking Pearson's correlations between the filtered time series within a defined time period for each pair of nodes [47].

### Time Windows for Temporal Network Construction

Dynamic networks are constructed by taking the filtered time series in temporal windows of 60 seconds and computing a 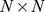 adjacency matrix of nodal correlations for each time window, where 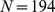 is the number of nodes. Each of these 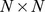 adjacency matrices represents the functional network over the 60 seconds in question. From this set of networks, we extract the edge weight time series by considering the correlation strength in each sequential network. We let 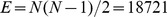 be the total number of edges between the 194 nodes and construct an 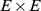 adjacency matrix **X**, where 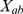 gives the Pearson correlation coefficient between the time series of edge weight for edges 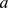 and 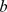. The entries of the 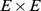 adjacency matrix represent pairs of edges with correlated weight time series [23].

We consider a range of temporal window lengths from 40 to 120 seconds and find that our results for hyperedge size and spatial distributions are robust to changes in window length in this range. Because the TR varies between the memory tasks and the rest and attention tasks, windows of equal time length include different numbers of data points in different segments of the experiment. To ensure this does not affect our analysis, we conduct an analysis with the number of data points per window held constant, and obtain very similar results (see Figure 1 in S1 Text).

### Hyperedge Construction

**Figure 2:**
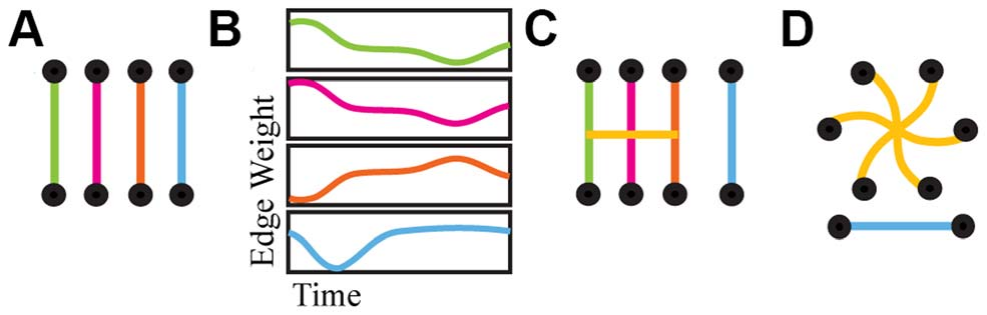
Hyperedge construction. A schematic illustration of the method used to identify hyperedges. We begin with a set of node-node edges (A) and their time series (B), of which three [green, pink and orange traces, (B)] exhibit strong pairwise temporal correlations. These edges are cross-linked (C) by temporal covariance in edge weight time series, and thereby form a hyperedge (D) of size three on six nodes. The final [blue] edge forms a singleton, an edge which is not significantly correlated with any other edges.

The cross-linked network structure, which contains information about groups of edges with similar time series (hyperedges), is extracted from the edge-edge correlation matrix **X** [23]. Fig. 2 provides a schematic illustration of the process of determining the cross-linked structure of a network. To exclude entries of **X** that are not statistically significant, we threshold **X** by evaluating the 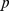-values for the Pearson coefficient 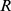 for each edge-edge correlation using a false discovery rate correction for false positives due to multiple comparisons [48]. If the 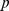-value for an entry 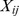 satisfies the false discovery rate correction threshold, we set 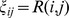 for our thresholded matrix 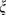. We set the thresholded entry of all other elements 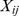 to zero. We binarize this thresholded matrix and obtain 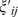, where
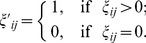
(1)


Each connected component in 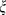 represents a hyperedge, a set of edges that have significantly correlated temporal profiles. The groups of nodes in Fig. 2(D) are examples of such connected components. A single hyperedge may include any number of edges between one (a singleton) and 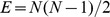 (the system size); these edges may be spatially clustered or at disparate locations throughout the brain. The set of all hyperedges defined in 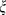 produces an individual hypergraph.

This hypergraph technique builds on recent trends in the wider field of network science. First, identifying groups of network edges that share similar properties, rather than the groups of nodes that have traditionally been the focus of community detection methods, has been recently shown to provide more intuitive representations of overlapping nodal communities and hierarchical structure [49]–[51]. Second, the idea of identifying functional groups based on the temporal patterns of their interactions has proven useful [51], [52]. Hypergraphs provide a straightforward method, both edge-based and intrinsically dynamic, of identifying and analyzing temporal patterns in network organization. In this work we focus on functional networks in the human brain, but the hypergraph-related diagnostics introduced below are easily generalizable to a broad variety of dynamic networked systems.

### Hypergraph Diagnostics

We use several methods to extract statistical features from individual hypergraphs and across the set of subjects.

#### Hyperedge size

We define the size, 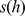, of a hyperedge 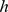, as the number of edges contained in it,
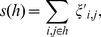
(2)where the sum is performed over the upper triangular elements of 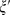, and 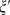 is the binarized edge-edge adjacency matrix defined above. Hyperedges with 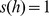 are singletons, which display no significant correlation between that edge and any other in the network. These singletons are excluded from further analyses. Additionally, we compute the cumulative hyperedge size distribution across all subjects in the study.

#### Hyperedge node degree

We define the hyperedge degree of a node to be the number of hyperedges that contain that node. We examine the hyperedge node degree distribution as a spatial distribution over the subjects as a group to understand characteristic hyperedge properties.

#### Co-evolution network

**Figure 3:**
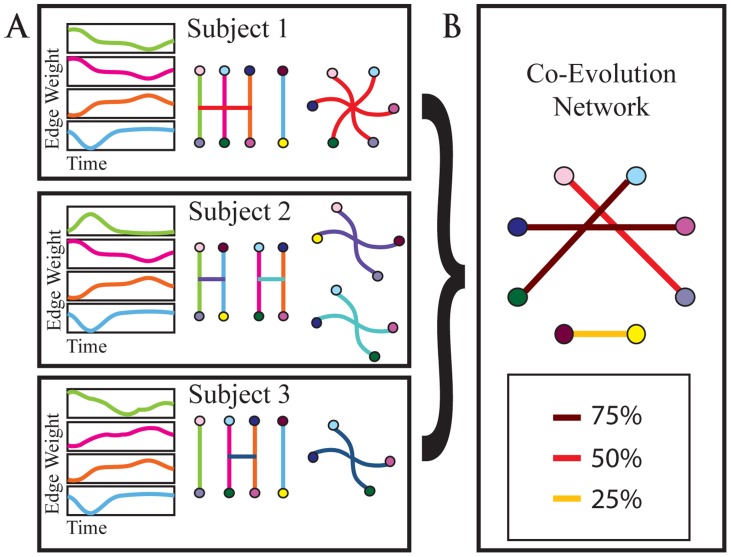
Schematic construction of the hyperedge co-evolution network. In (A), we analyze edge time series and group edges exhibiting similar temporal profiles into a hyperedge (as in Fig. 1). Here, node colors are used to indicate individual nodes and the edge color indicates distinct edges. We construct hypergraphs for each subject and find the matrix 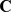 of probabilities that two nodes are in the same hyperedge over all subjects and hyperedges. In (B), this matrix is used to create a co-evolution network, where the weight for an edge connecting nodes 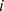 and 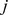 corresponds to the entry 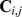.

We construct a “co-evolution network” to consolidate hypergraph results into a single graph that illustrates where hyperedges are most likely to be physically located over an ensemble of individuals. Fig. 3 illustrates a schematic of our construction. We begin by defining the matrix, 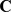, of probabilities that edges are included in a hyperedge over a set of hypergraphs. Again, nodes correspond to brain regions and connections correspond to inter-region associations, but here the weight of a connection joining nodes 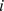 and 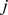 is the matrix entry 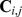. The resulting static network encompasses the dynamics of hyperedge activity, with connection weight corresponding to the probability that the two nodes are co-evolving over all of the hypergraphs considered. In later sections, we refer to co-evolution connection “strength,” which we define as the magnitude of the probability matrix entry corresponding to that connection.

### Task-Specific Classification

Previous work identified regions with task-specific activity in rest, attention, and memory tasks [30]. Further understanding of the regions that have a correlation structure unique to one task provides insight into network structure differences between tasks. To investigate the task-specific hyperedge structure, we first group hyperedges that exhibit a significantly higher correlation within one task into task-specific sets. If a hyperedge is significantly correlated in two or more tasks, it is excluded from the task-specific hypergraphs. The task-specificity of hyperedges is calculated by comparing the correlation within a single task to the correlation over the same time length with time points chosen randomly from other tasks. This permutation test uses a Bonferroni correction for false positives due to multiple comparisons [53]. Task-specific hypergraphs are then used to construct task-specific hyperedge size distributions, hyperedge node degree distributions, and co-evolution networks.

To quantitatively probe the differences in spatial organization of dynamic functional co-evolution networks for the four tasks, we investigate two summary metrics that show significant variation across tasks. Choice of these measures is primarily motivated by observed coarse differences in co-evolution network structure.

The first “length-strength” metric is the Pearson correlation coefficient, *R*, between the strength of a connection in the co-evolution network and Cartesian distance between the two nodes linked by the connection (physical length). The Cartesian distance is computed by taking the 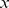, 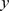, and 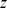 coordinates of each node and calculating the square root of the differences squared. The length-strength metric identifies a geometric property of the network, as well as a coarse estimate of the length of the strongest connections. Furthermore, connection length is related to network efficiency [54], [55], so differences in this measure could indicate varying levels of functional network efficiency corresponding to task states.

The second “position-strength” metric is the Pearson correlation coefficient, *R*, between the strength of the co-evolution network connection with the average anterior-posterior position of the two nodes. A measure of anterior-posterior position for each connection was found by taking the average 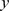 position of the two nodes in the connection. Identifying the location of strong co-evolution network connections along the anterior-posterior 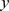 axis provides a measure of where hyperedges are physically present in task states. Both the structural core [33] and a dynamic functional core area, comprised of sensorimotor and visual processing areas [19], are located in the posterior, so nodes in these regions have negative 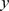 values. A larger negative position-strength value corresponds to a higher probability that hyperedges are active in these core areas.

The length-strength and position-strength metrics are evaluated for significance by comparing the correlation between length or position and connection strength to the same correlation performed on randomly chosen co-evolution connections. Again, the Bonferroni correction is performed to eliminate false positives due to multiple comparisons.

In Results, we discuss how these metrics reveal quantitative differences between task-specific networks. A more detailed analysis of the overlap between hyperedge co-evolution networks and relevant cognitive processing regions is also presented. In this analysis, we describe how delineated areas of higher hyperedge activity consistently correspond to recognized centers of task-specific activity.

### Null Models

In this analysis, we compare our results with two statistical null models based on measures for dynamic networks [22]. Hyperedges are formed from correlated edge time series; consequentially the null overall model randomly shuffles each edge time series over all experiments. This null model is designed to ensure that the hyperedges identified in our analysis can be attributed to the dynamics of the system, rather than some overall statistical property of the data set.

The other null test we perform, which we will refer to as the null within-task model, reorders each edge time series within each task, keeping tasks distinct. This is constructed in order to determine whether there are specific differences in the data between tasks.

## Results

We compile the results from the hypergraph analysis for each of the subjects and combine these results to obtain a size distribution, anatomical node degree distribution, and co-evolution network for the group. We then divide the data into task-specific hypergraphs and perform the previously mentioned analyses on the task-specific hypergraphs.

### Hypergraph Analysis and Statistics

**Figure 4:**
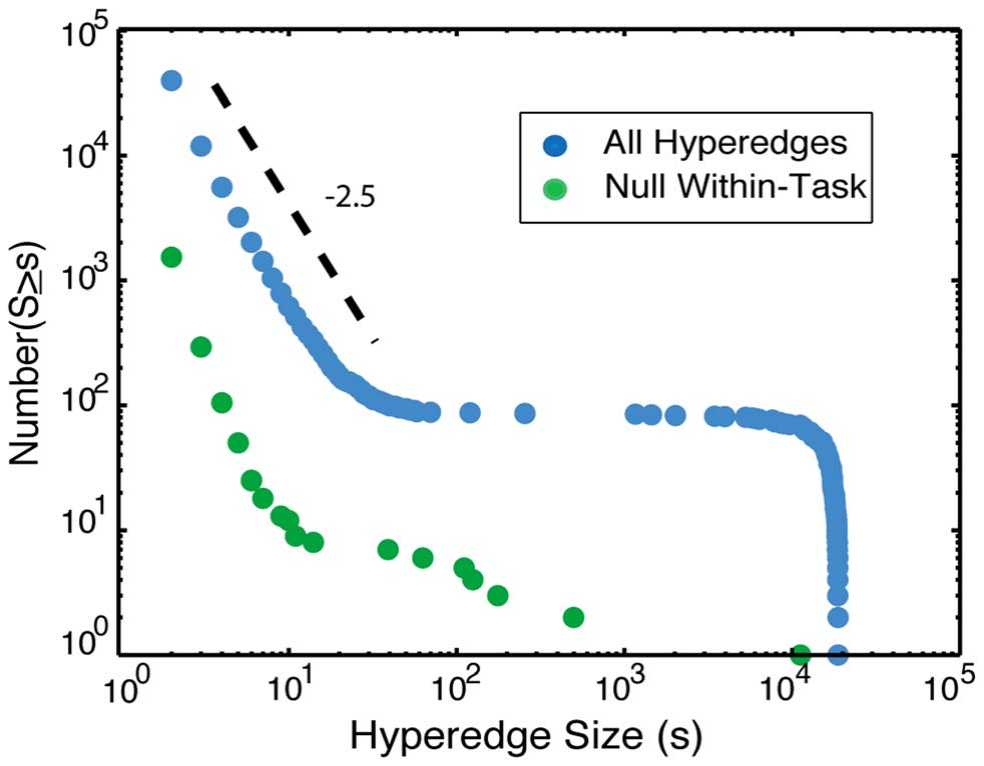
Hyperedge size distribution. In the cumulative frequency distribution of hyperedge sizes, the small hyperedges appear to roughly follow a power law with an exponent of approximately 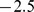, while the large group is concentrated near the maximum size. In the null overall model, there are no non-singleton hyperedges. Results for the null within-task model, where the data is shuffled within each task, are in green.

We construct a hypergraph for each individual and examine the cumulative distribution of hyperedge sizes (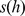 from Equation 2), shown in Fig. 4. There is a distinct break in the slope between two branches of the distribution occurring at a size of approximately 100 edges, which we use to distinguish between “large” and “small” hyperedges. The total number of small hyperedges appears to roughly follow a power law with an exponent of approximately 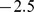. The number of large hyperedges peaks around the maximum size, with relatively few in the middle range from 100 to 1000 edges. In Fig. 4, the sharp drop off in the distribution at large hyperedge sizes reflects the system size limitation on hyperedge cardinality.

There is a distinct partition in all individual frequency versus sizes distributions; one or two “large” hyperedges (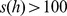), and many “small” hyperedges (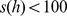) that peak at the smallest size. A subject with relatively small maximum hyperedge size has hundreds of edges in this largest hyperedge, as well as multiple “small” hyperedges. The corresponding hypergraph of a subject with a maximum hyperedge near the system size is strongly dominated by the largest hyperedge, which contains almost all edges in the brain.

The null overall model shuffles the data over all tasks. There are no hyperedges greater than size one, so the results from this null model are not depicted in Fig. 4. These singletons signify no significant correlation with other edges. As a result, we performed no further analysis on this null model. The fact that no significant hyperedges were found in the null overall model validates the statistical significance of our results.

The null within-task model shuffles the data but ensures that task data stays within the same task. The size distribution of hyperedges from the null within-task model is shown in Fig. 4. The shape of the two distributions is similar, although the null within-task model has fewer hyperedges in the large regime and there are more singletons than in the original data. This indicates there is co-evolution structure across tasks because this structure corresponds to changes in edge states between two or more tasks. For example, if groups of edges have an overall high correlation in one task and a significantly lower correlation in another, it would induce a hyperedge across the tasks regardless of how the within-task time series are shuffled.

**Figure 5:**
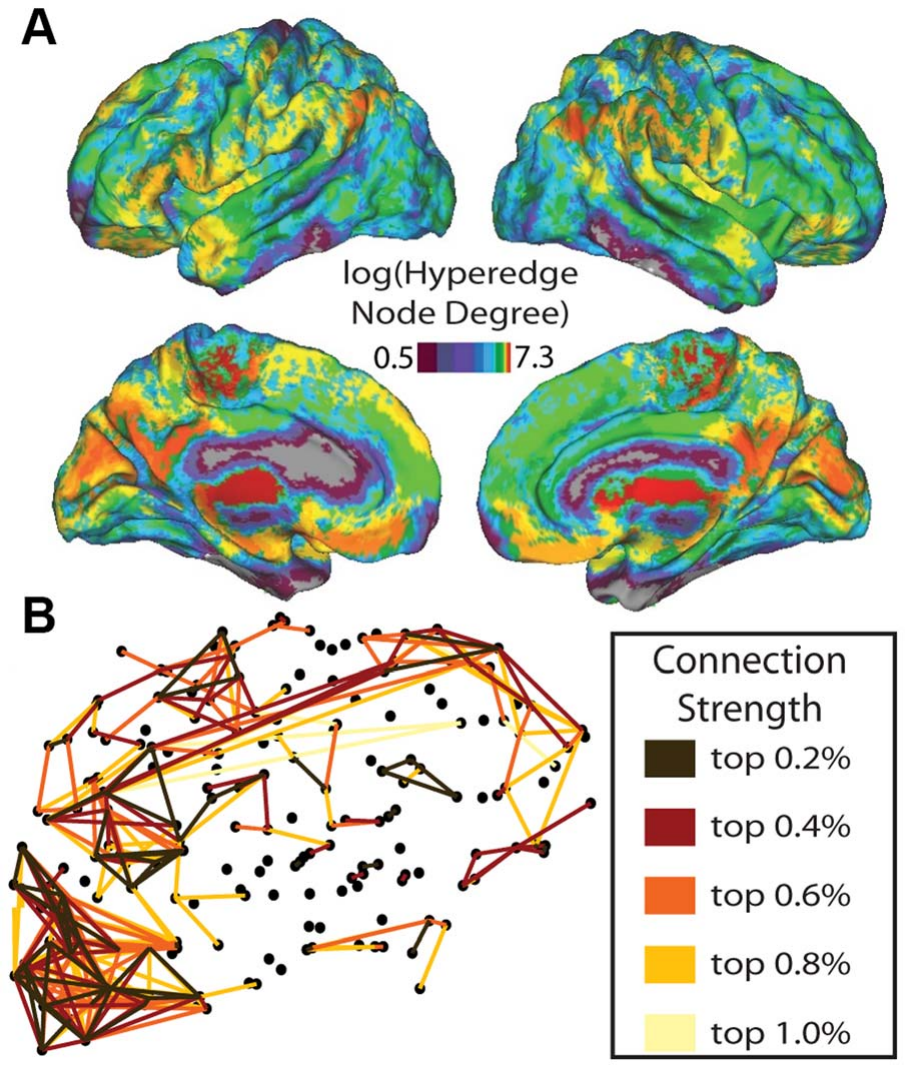
Hyperedge node degree and co-evolution network. In (A), we show hyperedge node degree on a natural log scale. The cumulative number of hyperedges at each node over all individuals is plotted on the brain, where higher values at a node correspond to more hyperedges that include the node. (B) depicts a sagittal view of the co-evolution network. The edge strength represents the probability that the edge will be in a hyperedge over all individuals. Edge color corresponds to threshold percentage value, where only the top 1% of co-evolution probabilities are shown. Within this 1%, brown connections correspond to the highest 0.2% of probabilities, red connections correspond to 0.2% to 0.4%, orange connections correspond to 0.4% to 0.6%, gold connections correspond to 0.6% to 0.8%, and yellow connections correspond to 0.8% to 1%.

Examining the cumulative hyperedge size distribution provides information about the network topology but does not supply descriptive spatial information. Next, we quantify which anatomical locations in the brain participate in hyperedges, identifying differential roles in task-induced co-evolution. Fig. 5A depicts the hyperedge node degree on a natural log scale. The densest regions are located in posterior portions of the cortex, primarily in visual areas, while a second set of dense regions is located in the prefrontal cortex.

We construct a co-evolution network, as illustrated schematically in Fig. 3, where connection weight corresponds to the probability that two nodes participate in the same hyperedge. In Fig. 5B we present this co-evolution network over all individuals and all tasks. The graph includes sparse long-range connections between regions that are densely connected. Within the strongest 1% of connections, the high degree of bilateral symmetry indicates that corresponding nodes in the left and right hemispheres have a high likelihood of being placed together in a hyperedge. Dense areas of the graph include primary visual areas, portions of prefrontal cortex, and primary motor cortex.

### Task-Specific Hyperedges

**Figure 6:**
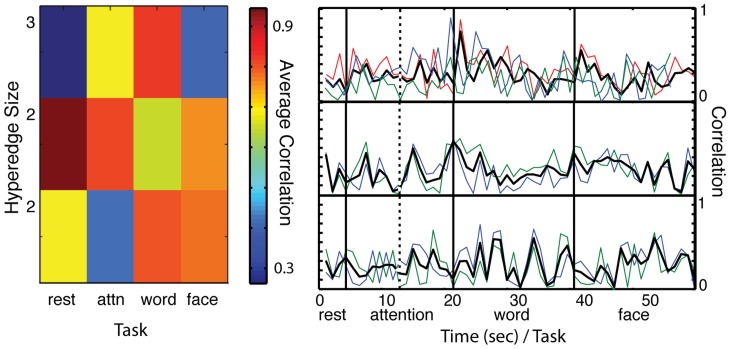
Task-specific hyperedges. **Left:** Average hyperedge correlation in each task for three hyperedges (where hyperedges with small sizes are chosen for illustrative purposes). **Right:** Correlation (absolute value) time series for the same three hyperedges. The colored lines represent each edge, while the black line is the average edge time series. Each time point represents the static network over 60 seconds, and the attention task is broken into two sections because two separate iterations of the same task were combined in this analysis. These results display the task-specificity of hyperedges, where significant correlations in the hyperedge are restricted to one task. For example, the first hyperedge is word-specific because there is a much stronger average correlation in the word task than in any other task.

The hypergraph algorithm groups together edges with significantly similar temporal behavior. However, this basic classification does not distinguish whether the correlation is present throughout the edge time series, or whether highly correlated sections of the time series drive the selection. We compute the average within-task edge correlation for each hyperedge and find that in some cases, strong edge correlation spans the tasks, while in other hyperedges, a strong correlation between edges within one task drives the hyperedge. An example of this task-specific correlation structure can be seen in Fig. 6. In the average within-task correlation on the left, there is a stronger average correlation in the word memory task than in any other task. Furthermore, the edge time series in the first hyperedge indicates it is driven mainly by a correlation within the word memory task.

To investigate this further, we construct task-specific co-evolution networks, composed of hyperedges with significantly stronger average correlation in one task than the others (see Methods). To identify these task-specific hyperedges for each task, we perform a permutation test on the edge weight time series, as described in Methods, and compare the total correlation within the task to the expected values. If a hyperedge displays significant edge correlation (determined by the Bonferroni correction on the 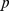-values from the permutation test) in only one task, we label it as a task-specific hyperedge. Hyperedges with two or more tasks exhibiting significant correlation are not included in the task-specific hypergraphs.

**Figure 7:**
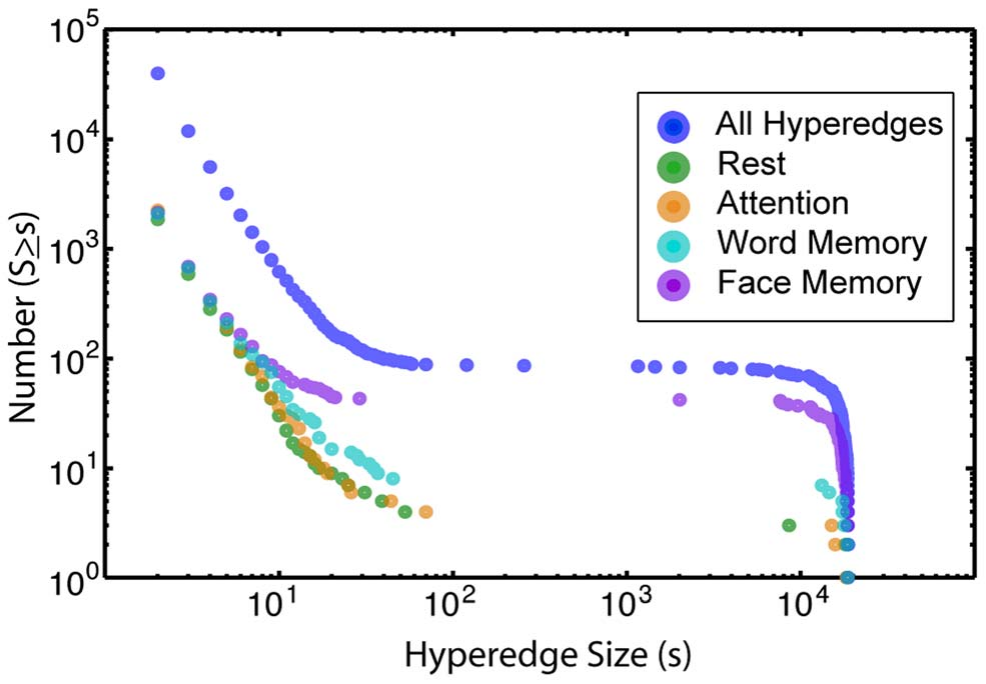
Task-specific hyperedge size distributions. Cumulative frequency distribution as a function of hyperedge size for all task-specific groups. The results are compared to the overall distribution of hyperedges (dark blue), previously illustrated in Fig. 4. There are fewer large hyperedges attributed to attention and rest tasks, while the memory tasks have a greater number of large task-specific hyperedges.

Fig. 7 illustrates the size distributions of all the task-specific results alongside the overall hyperedge size distribution. The sizes and spatial distributions of single task-driven hyperedges vary across tasks and incorporate significant information about functional network organization with respect to changing cognitive states. Attention has the greatest number of task-specific hyperedges, followed by face memory, word memory, and rest. In the small regime, the tasks follow a similar distribution. There are fewer large attention and rest hyperedges, while the face memory task closely mimics the overall distribution. The distinction in the distributions indicates that the tasks can be characterized by differing complexities of edge co-variations.

**Figure 8:**
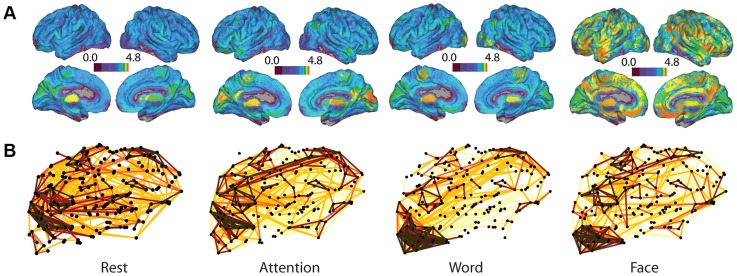
Task-specific co-evolution networks and hyperedge node degrees. (A): Distribution of task-specific hyperedge node degree on the brain. Here, the log of the total number of hyperedges containing each node is represented on the brain. The color scale represents the log of hyperedge node degree as in 5A, although here the range of values is from 0 to 4.8. (B): Co-evolution networks for each task. Edge strength corresponds to the probability that a hyperedge will contain the edge over all individual hypergraphs. Color represents a threshold in percentage value, with the scale given in Fig. 5B, and the top 1% of co-evolution probabilities are shown. Once again, the top 2% of probabilities are brown, red indicates the top 0.2% to 0.4% of connections, orange indicates the top 0.4% to 0.6% of probabilities, gold indicates the top 0.6% to 0.8% of probabilities, and yellow indicates the top 0.8% to 1% of probabilities.

**Figure 9:**
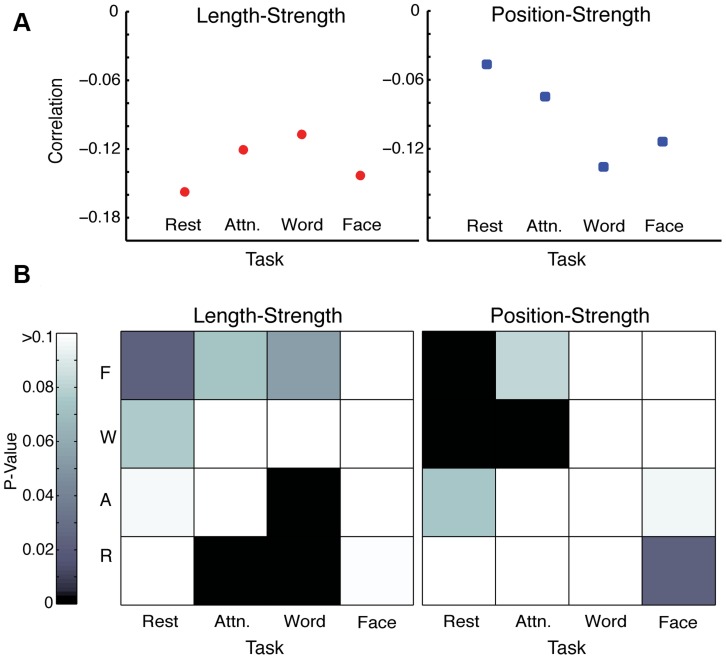
Task-specific network statistics. Values for the position-strength metric (blue) and the length-strength metric (red) for the four tasks are depicted in (A). (B) shows 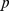-values for the pairwise statistical permutation test between tasks, where black denotes a significant value after a Bonferroni correction for multiple comparisons. Values are obtained for length-strength and position-strength metric. For example, on the 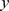 position plot in (B), attention-word is significant. Referring back to (A), we see that this implies the difference in the 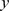 position-strength correlation between the attention and word tasks is statistically significant.

The spatial distributions of hyperedge node degree in each task, along with task-specific co-evolution networks, are shown in Fig. 8. The rest hypergraph has the least activity in posterior regions of the cortex, both in the hyperedge node degree plot and co-evolution network. In the attention network, long connections connecting the front and back of the brain distinguish it from the rest network. Furthermore, the concentration in the occipital lobe is larger in the memory co-evolution networks than in the rest or attention networks. We characterize these observed differences with two statistics, which are described in more detail in Methods. The length-strength metric is a correlation between connection length and strength in the co-evolution network. The position-strength metric is a correlation between connection position (anterior-posterior) and strength. The results of this analysis over the full unthresholded co-evolution network are in Fig. 9. All correlation values are negative, indicating that, in all tasks, stronger connections in the co-evolution network are located in posterior portions of cortex and are physically shorter.

We compare these values across tasks by performing pairwise permutation tests to determine which networks have statistically different properties. Fig. 9 depicts the 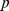-values from these tests, where the horizontal axis represents the statistic being tested and the vertical axis corresponds to the task being tested against. The black squares in this figure represent significant values, which are summarized in the following list:

1. The rest task has a significantly less strong position-strength correlation than the word and face memory tasks. This confirms the observation that the rest co-evolution network is less likely than the memory networks to have strong connections in posterior regions of the cortex.
2. The attention task is less strongly correlated than the word memory task, as measured by the position-strength metric and the rest task in terms of the length-strength metric. Thus, the attention co-evolution network is less likely than word memory to have strong connections in the posterior, and less likely than the rest network to have strong connections that are short.
3. The word memory task has a weaker length-strength correlation than the rest and attention tasks. Thus, strong connections in the word memory co-evolution network are less likely be short than they are in attention and rest networks.

These results delineate significant differences in co-evolution network structure between the tasks, confirming that the hypergraph analysis is a useful method for distinguishing between task states. Additional features of the task-specific co-evolution networks are described in more detail below.

#### Rest

Rest-specific hyperedges are primarily represented in the “small” range of the size distribution in Fig. 7. Although it is difficult to distinguish in Fig. 7 due to the logarithmic scale, the rest task also has the lowest number of task-specific hyperedges. Consequently, its spatial hyperedge node degree distribution in Fig. 8A has the lowest overall magnitude across task states. The areas with the highest degree of hyperedge activity are in the posterior portions of the brain, a configuration that is consistent across tasks. This suggests there is an underlying pattern of hyperedge generation centered in the occipital lobe.

The rest-specific co-evolution network is highly clustered in the most probable 0.2% of co-evolution pairs, as visualized in Fig. 8B. High probability clusters occur in areas including the inferior parietal lobule, superior frontal gyrus, precuneus, and posterior cingulate cortex. Although the rest network displays clustering at this highest threshold of probability, lower thresholds show very little structure; the top 1% of connections shown in Fig. 8B is far more randomized in rest than in the other task-specific co-evolution networks. There is relatively little lateral symmetry and few visible “core” areas with high hyperedge node degree.

The negative length-strength correlation for connections in the co-evolution network is significantly stronger for the rest task than the word memory task. This indicates that the strongest connections in the rest-specific co-evolution network are short, reflecting the initial observations in Fig. 8B. The rest co-evolution network also has the smallest negative correlation between connection position and strength, which the permutation test (Fig. 9B) confirmed to be significantly smaller than the word or face memory tasks. This means that the strongest rest-specific hyperedges are less likely to be located in the posterior of the brain than the strongest hyperedges specific to either memory task, a result again consistent with Fig. 8B.

#### Attention

Overall, there are more hyperedges associated with attention than any other task, although this is difficult to visualize in Fig. 5. The attention-specific hypergraph consists almost exclusively of small hyperedges. This lack of large hyperedges may account for the increased disorganization in the co-evolution structure at lower probability thresholds observed in both rest and attention co-evolution networks in Fig. 8B.

The spatial hyperedge node degree distribution for the attention task (Fig. 8A) appears qualitatively similar to the rest task, with a few areas of increased degree in the occipital lobe, and with overall larger hyperedge node degree values corresponding to the greater overall number of attention-specific hyperedges compared to rest.

The co-evolution structure specific to the attention task (Fig. 8B) has a higher degree of bilateral symmetry than the rest network, and has fewer strong connections in the occipital lobe than either memory task. There are multiple prefrontal cortical regions that are likely to cohesively evolve with several other nodes. Regions of high clustering in the most probable threshold include the lateral parietal and occipital lobes, the superior frontal cortex, and dorsal parietal cortex.

Numerous strong connections between rostral and caudal brain regions are another feature of the attention-specific co-evolution network. The negative length-strength correlation in the attention co-evolution network is significantly less strong than in the rest task, consistent with the observation that the attention network has strong connections that reach across the brain (Fig. 8B). Additionally, the attention task has a significantly weaker position-strength correlation than the word memory task, likely driven by the strong attention co-evolution connections in the prefrontal cortex.

#### Memory for words

The word memory-specific hyperedge size distribution includes more large hyperedges than rest or attention, although it is not as close to the overall distribution as the face memory distribution.

The spatial hyperedge node degree distribution for the word memory task has high node degrees in similar brain areas to the other task-specific distributions. There is a marked increase in node degree of regions in the parietal lobe from rest and a decrease in degree of regions in the occipital lobe from attention (seen in Fig. 8A).

In the word memory co-evolution network in Fig. 8B, the strongest connections are highly clustered in the occipital or frontal lobes, with few connections to nodes in between, while the strength and number of bilateral links is diminished compared to the attention task. The negative length-strength correlation of connections in this network is the weakest for the word memory co-evolution network, and significantly weaker than in the rest or face memory tasks. As in the attention task, this is consistent with the many connections between the occipital and frontal lobes visible among the strongest links in the word memory co-evolution network (Fig. 8B).

#### Memory for faces

There are more large hyperedges significantly correlated in the face memory task than any other task-specific group. The task-specific size distribution closely resembles the overall distribution in the large regime, indicating that a significant portion of all large hyperedges are driven by correlations in the face memory task.

The face memory-specific hyperedge node degree values are consistently the largest across the brain. This is primarily due to the many large hyperedges specific to the face memory recognition task. In the word memory and attention degree distributions, there are areas of higher hyperedge node degree in the parietal lobe and occipital lobe, respectively, but the face memory degree distribution is more evenly dispersed over the brain.

The structure of the face memory-specific co-evolution network, shown in Fig. 8B, is most dense in the occipital lobe, consistent with the visual nature of the task. There are several strong connections from the occipital lobe to other brain regions, specifically in the prefrontal cortex and frontal lobe. While the structure looks similar to that of the word memory co-evolution network, the strong cluster of face memory co-evolution connections in the occipital lobe has fewer strong connections and less nodes involved overall than the corresponding word memory co-evolution network cluster, but more strong connections to a few particular nodes. Compared to word memory, the face memory-specific network also displays fewer strong connections in the frontal lobe but more strong connections among regions in the dorsal attention network. In addition to the properties discussed in previous sections, the face memory co-evolution network has a strong negative position-strength correlation, indicating that the strongest connections tend to be in the posterior of the brain.

## Discussion

Progress in understanding functional brain network topology provides significant insight into broad neuroscience questions regarding the brain's organization and ability to effectively transition between cognitive states. Quantifying complex network dynamics in the brain will further understanding in these areas and has promising applications to behavioral adaptation and learning [18], [19], [21]. We apply hypergraph analysis, a tool from dynamic network science, to functional brain imaging data in order to determine co-evolution properties of the brain as subjects perform a series of tasks. A previous application of this method to neuroscience uses hypergraphs to analyze how functional network structure changes over a long term learning task [19]. The learning experiment considers hypergraphs constructed over 6 weeks of training while subjects acquire a new motor skill, while our analysis compares hypergraphs over three different tasks performed within an interval of hours. Our analysis shows that hypergraphs are a useful tool for investigating shorter time scales and differentiating between task-specific networks.

Instead of analyzing the time-dependent behavior of groups of nodes, the hypergraph investigation considers the edge weight time series, where edges with statistically significant similarities in their temporal profiles are grouped into hyperedges. This approach is advantageous because it considers all edges, regardless of correlation strength, unlike previous methods which focus exclusively on strong correlations [30], [56]. The use of a data-driven analysis also allows us to investigate the dynamic changes in brain function over a series of tasks without prior assumptions of the structure of the connectivity network. This is a significant advantage over methods that characterize task states based on their differences with respect to the rest network [3], [4]. A comparison between the hypergraph analysis and these methods in a future analysis could reveal how the concentration of hyperedges varies in known task-positive or task-negative areas and determine whether this variation has an effect on task performance.

### Hypergraph Statistics and Structural Metrics

We demonstrate the existence of hypergraph structure in functional brain dynamics and statistically characterize the hyperedge distributions in comparison to appropriate null models. Shuffling the time series over all time produces no significant hyperedges, while shuffling within each task results in a size distribution that resembles the overall size statistics in shape, but with far fewer hyperedges. The distinct differences between the two null models and our results based on the original time series establish the significance of our findings. Furthermore, the existence of hyperedges after the within-task shuffling indicates the presence of activity in some edges that is differentiated between tasks. Since there are fewer large hyperedges after the within-task shuffling, we can also confirm that there are hyperedges caused by edge dynamics within tasks. This work primarily concentrates on hyperedges correlated within a particular task, but future analyses to understand the properties of hyperedges that are grouped due to other general properties would supplement our results.

The hyperedge size distribution is comprised of “small” and “large” hyperedges, where the size distribution of the small hyperedges follows a power law and the large hyperedges peak at the system size. We explore the overall spatial hyperedge distribution by constructing a hyperedge node degree plot, and find that the majority of the most densely connected nodes lie in the posterior portions of the brain. To better observe spatial hyperedge properties, we develop a co-evolution network, where connection weights correspond to the probability that a hyperedge will include the connection. The top 1% of connections in the network with the highest probability of inclusion in a hyperedge are most concentrated in the occipital lobe and prefrontal cortex. These are expected areas of hyperedge concentration, consistent with the visual nature of the tasks, as well as the coordination of quick decision making and the selection of specific motor responses.

### Task-Specificity and Anatomical Placement

We find there are hyperedges that are more correlated in one task and hyperedges that have a distinct profile across the tasks. Our results suggest that edges with a high probability of inclusion in task-specific hyperedges are often found in previously identified brain areas associated with the corresponding tasks, as discussed in detail below, confirming that the approach captures relevant information about task networks. In some cases, brain regions expected to show strong co-variation in a certain task are not included among the strongest connections of that task-specific co-evolution network; we also discuss examples of this in detail below. Repeating the analysis and grouping hyperedges that are significantly correlated in two tasks might lend insight into whether brain systems relevant to a certain task contain hyperedges that are correlated in another task and thus are rejected from our task-specific analysis.

In all tasks, stronger connections in the co-evolution network tend to be located in posterior portions of cortex and to be physically shorter. The higher probability of posterior edges to be included in hyperedges is consistent with the identification of a core set of highly structurally connected regions centered in the posterior of the brain, thought to play an important role in integrating large-scale functional connectivity [19], [33]. The tendency of strong connections to be physically shorter suggests high efficiency in task-specific co-evolution networks. This may reflect efficient wiring properties associated with minimal wiring for rapid processing and low energy expenditures found in structural brain networks and shared by some other biological and technological networked systems [57].

#### Rest

Resting-state brain activity contains correlated patterns that comprise a default mode network, a system that is engaged during internal cognition [58], [59]. Certain brain regions active at rest are consistently deactivated during goal-oriented tasks, indicating that they comprise a functional mode that is rest-specific [1].

Our result that rest has fewer specific hyperedges than the attention or memory tasks could be a result of the specificity of correlated resting state regions, or a simplicity intrinsic to resting state function that does not necessitate more concerted efforts involving numerous brain regions [11]. In addition, we see a relative randomization and asymmetry in the spatial co-evolution distribution of rest-specific hyperedges, as well as a relative lack of long, strong connections; these results may correspond to a diminished need for efficient processing in a task-free environment.

Dense areas of the co-evolution network with high probabilities of being in rest-specific hyperedges include brain regions traditionally associated with the resting state. The inferior parietal lobule, superior frontal gyrus, precuneus, and posterior cingulate cortex have been identified as integral components of the default mode network; in addition, the posteromedial cortex, which includes the precuneus and posterior cingulate cortex, plays an important role in awareness [60]–[62].

#### Attention

Two attention systems exist in the human brain: a “top-down” network controls goal-directed attention, while a “bottom-up” group of brain regions detects and orients attention to relevant sensory stimuli that are generally novel or unexpected [63], [64]. Our task probes the former, as subjects are asked to focus on repetitive stimuli in a controlled environment. This requires an “executive control network,” a bilateral dorsal system that governs guided attention and working memory [65]. The relatively high degree of bilateral symmetry and the dorsal concentration of connections observed in the attention-specific co-evolution network suggests a higher probability for connections within this executive control network to co-evolve with other edges during the attention task.

Specifically, we observe regions of high clustering among the strongest connections in the attention-specific co-evolution network in the lateral parietal and occipital lobes, superior frontal cortex, and dorsal parietal cortex, areas known to be involved in attention networks. Parietal and frontal areas are involved in attention control and localization, specifically in visual attention tasks [63], [66]. Activation of the superior frontal cortex occurs in attention tasks, especially those that involve a shift to peripheral locations in the visual field [67], [68]. The dorsal parietal cortex also performs a central role in the executive control network: patients with lesions in the dorsal parietal cortex have shown significant impairment in goal-directed attention tasks [69].

Strong connections in the attention co-evolution network are more likely to be long than those in rest, corresponding to the high probability that long rostral-caudal edges will be included in hyperedges (visible in Fig. 8B). This may reflect a greater need for coordination between prefrontal executive control regions and regions in the occipital lobe during the attention task. In addition, strong attention-specific co-evolution connections are less likely to be located in the posterior of the brain than those specific to word memory; this could indicate that the attention task state has less reliance on core visual regions than the word memory task state.

#### Memory for words

Our results for the word memory-specific and the face memory-specific hypergraphs were similar in several ways. Both displayed many more “large” hyperedges than the rest or attention tasks, suggesting that some aspect of the memory tasks requires dynamically coherent evolution over much of the brain. We speculate that this variation in the task-specific size distributions may correspond to the cognitive complexity demanded by the tasks, with the more involved memory tasks requiring more coordination between different cognitive networks and functions, and therefore producing more large hyperedges. This possibility could be further tested by examining hyperedge size variation across tasks specifically designed to vary in complexity.

Visual orthographic and face processing have a common reliance on central vision [70] and share neural circuitry [71]. The resemblance of the co-evolution networks for the two tasks, especially when compared with the very different graph structure of the attention and rest networks, indicates a similarity in the hypergraph representation of the memory tasks. This in turn signifies a correspondence in brain dynamics specific to memory. The task-specific analysis identifies hyperedges that show a significant correlation in only one task, so there is no overlap in these co-evolution networks.

Existence of a dedicated visual word processing network has been a topic of frequent discussion in neuroscience. The visual word form area (vWFA), located in the occipito-temporal cortex, is consistently activated by orthographic stimuli [72] and is invariant to changes in case, size, font, or type of visual stimulation [73], [74]. The vWFA has also been shown as functionally linked to the dorsal attention network in resting state fMRI data, indicating that it fulfills a complex cognitive role [75].

In the word memory-specific co-evolution network, the vWFA is highly connected, but there is minimal strong structure in dorsal attention areas, which we would expect to see in a functional connectivity analysis [75]. This can be explained by our methodology of selecting task-specific hyperedges. If edges in the dorsal attention network have similar co-evolution properties within the word memory and attention tasks, they will not be identified as task-specific edges.

#### Memory for faces

Face recognition in humans requires a complex network distributed throughout the visual cortex that includes extended connections branching to other cortical regions [76]. The majority of visual processing occurs in the occipital lobe, located in the posterior of the brain. Functional MRI studies have identified multiple regions in the occipital cortex that respond more strongly to faces than other visual stimuli, indicating that the cognitive processes involving facial recognition are highly specialized [77], [78]. The especially dense concentration of connections in the occipital lobe at the highest probability levels of the face memory-specific co-evolution network is consistent with this.

The face perception system is composed of multiple bilateral regions; the lateral symmetry observed in the face memory-specific co-evolution network is consistent with this structure [76]. An aspect of the co-evolution network that breaks this symmetry is the right fusiform gyrus, which is strongly connected to other areas in the occipital lobe by high probability co-evolution pairs. A region in the fusiform gyrus, the fusiform face area (FFA), has been found to be selectively active in whole human facial perception, and the right FFA in particular has been found to have the most salient response to faces, with damage to the region severely impairing face recognition [79], [80]. The high probability of co-evolution between the right fusiform gyrus and other regions in this task-specific hypergraph is consistent with our expectation that regions involved in the memory of faces in particular (as opposed to words) are most likely to be included in face memory-specific hyperedges.

The co-evolution networks for both memory tasks show a significantly higher hyperedge probability in visual areas than the attention and rest tasks, and the differences in structure indicate that the hypergraph representation of memory tasks is significantly different from rest or attention. The marked differences in hyperedge statistics between task states in our task-specific analysis suggest hypergraphs as a measure of functional network changes due to task states. With measures derived from the hyperedge analysis, we can begin to quantitatively probe the mechanisms of functional switching between tasks and gain insight into how distinct features of the network evolve in synchronized patterns.

### Methodological Considerations

Because they consider both strong and weak edges with no thresholding, hypergraphs are well-suited for identifying groups of brain regions that, for example, initially have uncorrelated activity but become more correlated in synchrony (or vice-versa), as we expect task-associated cognitive networks to do as a result of switching between tasks. In order to extract these dynamic patterns, the hypergraph technique considers strong and weak edges equally, ignoring any offset between the average correlation strengths of different edge time series. This is intended to provide a complementary method to the common thresholding approach of separating or ignoring network edges with correlation strengths weaker than some critical value [30], [56]. Since weak edge connectivity has been shown to contain functionally relevant and predictive information in various contexts, retaining these edge weights is desirable [44], [81], [82]. There is also evidence that mean edge correlation values can be driven by non-biological artifacts such as head motion, even after applying standard motion-correction techniques [20]; by remaining indifferent to edge weight offsets, a hypergraph analysis avoids this concern.

In applications where the overall correlation strength of network edges is nevertheless important, it may be useful to supplement the dynamic information given by a hypergraph analysis with a measure that retains this edge weight information. Efforts to make quantitative comparisons between the hypergraph analysis and other dynamic graph theoretical methods in the context of the human brain are ongoing. We are currently investigating whether dynamic community detection on weighted brain networks, a node-based analysis which relies on edge correlation strength, provides complementary information to the hypergraph analysis.

Because we choose a linear measure to compute correlations between edge weight time series, our analysis as presented here does not account for time lag in these correlations. However, our framework could be extended to nonlinear measures that include time-lag information.

It is important to note that our method of computing a dense matrix of edge-edge correlations and thresholding according to significance does not necessarily identify direct conditionally-dependent correlations between time series, or correlations that represent the underlying structural connectivity of the brain. As with any method that infers a network structure from correlation data simply by thresholding, we expect many of these correlations to be indirect. For example, a significant correlation between two edge weight time series may occur because both edges are being controlled by a third, more central edge – and not because the two edges are directly connected either causally or structurally. In this sense, the edge-edge correlation structure does not capture relations that necessarily reflect the underlying control structure or the physical architecture of the brain. Our hyperedge analysis moves the focus away from such indeterminate dyadic relationships, considering only groups of all edges that share similar dynamic patterns without any intra-group organization or structure.

It is also possible, as in any fMRI analysis, that edge-edge correlations arise from task-induced indirect drivers, such as visual stimuli. Two regions that are both activated by a visual stimulus may show strong functional connectivity with one another in a single time window. Moreover, such regions may show similar changes in functional connectivity over time if their activation profiles to the stimulus evolve similarly during the experiment. As with any measurement of functional connectivity based on the Pearson correlation coefficient [83], a common and robust measurement of functional connectivity, such indirect drivers of functional connectivity are not distinguished from other more direct drivers of communication or interaction.

Throughout this work, we observe a significant amount of individual variability in the hypergraph properties of interest. In this manuscript, we have completed a group-level analysis and focused on investigating task-related differences in hypergraph structure. However, individual variability may be related to differences in cognitive ability and provide additional insight into the role of hyperedges in task performance, which is a topic of future research.

### Final Remarks

In this paper, we use hypergraph analysis to identify significant co-evolution between brain regions in task-based functional activity and develop new tools to summarize the spatial patterns of these co-evolution dynamics over the group of subjects. By isolating task-specific hyperedges, we quantify significant differences between the spatial organization of co-evolution dynamics within different tasks. This hypergraph analysis adds a crucial perspective to previous treatments of task-based brain function, describing temporal similarities between spatially segregated neural circuits by specifically examining the organization of connections that co-evolve in time. It provides a promising approach for understanding fundamental properties of task-based functional brain dynamics, and how individual variation in these properties may correspond to differences in behavior and task performance.

## Supporting Information

S1 Text **Supplementary methodological information.** Discussion of the effects of time window selection and brain region size on the results, with accompanying figures.
(PDF)

## References

[1] Raichle ME, MacLeod AM, Snyder AZ, Powers WJ, Gusnard DA, et al (2001) A default mode of brain function. Proceedings of the National Academy of Sciences 98: 676–682. 10.1073/pnas.98.2.676 PMC14647 11209064

[2] Damoiseaux JS, Rombouts SA, Barkhof F, Scheltens P, Stam CJ, et al (2006) Consistent resting-state networks across healthy subjects. Proceedings of the National Academy of Sciences 103: 13848–13853. 10.1073/pnas.0601417103 PMC1564249 16945915

[3] Fox MD, Snyder AZ, Vincent JL, Corbetta M, Van Essen DC, et al (2005) The human brain is intrinsically organized into dynamic, anticorrelated functional networks. Proceedings of the National Academy of Sciences 102: 9673–9678. 10.1073/pnas.0504136102 PMC1157105 15976020

[4] Hampson M, Driesen N, Roth JK, Gore JC, Constable RT (2010) Functional connectivity between task-positive and task-negative brain areas and its relation to working memory performance. Magnetic Resonance Imaging 28: 1051–1057. 20409665 10.1016/j.mri.2010.03.021 PMC2936669

[5] Friston KJ (2011) Functional and effective connectivity: a review. Brain Connectivity 1: 13–36. 22432952 10.1089/brain.2011.0008

[6] Bassett DS, Meyer-Lindenberg A, Achard S, Duke T, Bullmore E (2006) Adaptive reconfiguration of fractal small-world human brain functional networks. Proceedings of the National Academy of Sciences 103: 19518–19523. 10.1073/pnas.0606005103 PMC1838565 17159150

[7] Bullmore E, Sporns O (2009) Complex brain networks: Graph theoretical analysis of structural and functional systems. Nature Reviews Neuroscience 10: 186–198. 19190637 10.1038/nrn2575

[8] Mennes M, Kelly C, Zuo XN, Di Martino A, Biswal BB, et al (2010) Inter-individual differences in resting-state functional connectivity predict task-induced BOLD activity. NeuroImage 50: 1690–1701. 20079856 10.1016/j.neuroimage.2010.01.002 PMC2839004

[9] Cole MW, Reynolds JR, Power JD, Repovs G, Anticevic A, et al (2013) Multi-task connectivity reveals flexible hubs for adaptive task control. Nature Neuroscience 16: 1348–1355. 23892552 10.1038/nn.3470 PMC3758404

[10] Mennes M, Kelly C, Colcombe S, Castellanos FX, Milham MP (2013) The extrinsic and intrinsic functional architectures of the human brain are not equivalent. Cerebral Cortex 23: 223–229. 22298730 10.1093/cercor/bhs010 PMC3513960

[11] Cole MW, Bassett DS, Power JD, Braver TS, Petersen SE (2014) Intrinsic and task-evoked network architectures of the human brain. Neuron 83: 238–251. 24991964 10.1016/j.neuron.2014.05.014 PMC4082806

[12] Hutchison RM, Womelsdorf T, Allen EA, Bandettini PA, Calhoun VD, et al.. (2013) Dynamic functional connectivity: promise, issues, and interpretations. NeuroImage: 360–378. 10.1016/j.neuroimage.2013.05.079 PMC3807588 23707587

[13] Ekman M, Derrfuss J, Tittgemeyer M, Fiebach CJ (2012) Predicting errors from reconfiguration patterns in human brain networks. Proceedings of the National Academy of Sciences 109: 16714–16719. 10.1073/pnas.1207523109 PMC3478635 23012417

[14] Doron K, Bassett DS, Gazzaniga MS (2012) Dynamic network structure of interhemispheric coordination. Proceedings of the National Academy of Sciences 109: 18627–18628. 10.1073/pnas.1216402109 PMC3503189 23112199

[15] Siebenhühner F, Bassett DS (2013) Multiscale Analysis and Nonlinear Dynamics: From Genes to the Brain, Wiley & Sons, chapter Multiscale Network Organization in the Human Brain.

[16] Cohen JR, Gallen CL, Jacobs EG, Lee TG, D′Esposito M (2014) Quantifying the reconfiguration of intrinsic networks during working memory. PLoS ONE 9: e106636. 25191704 10.1371/journal.pone.0106636 PMC4156328

[17] Monti RP, Hellyer P, Sharp D, Leech R, Anagnostopoulos C, et al (2014) Estimating time-varying brain connectivity networks from functional MRI time series. NeuroImage S1053–8119: 00616–00618. 10.1016/j.neuroimage.2014.07.033 25107854

[18] Bassett DS, Wymbs NF, Porter MA, Mucha PJ, Carlson JM, et al (2011) Dynamic reconfiguration of human brain networks during learning. Proceedings of the National Academy of Sciences 108: 7641–7646. 10.1073/pnas.1018985108 PMC3088578 21502525

[19] Bassett DS, Wymbs NF, Romback PM, Porter MA, Mucha PJ, et al (2013) Task-based core-periphery structure of human brain dynamics. PLoS Computational Biology 9: e1003171. 24086116 10.1371/journal.pcbi.1003171 PMC3784512

[20] Bassett DS, Yang M, Wymbs NF, Grafton ST (2014) Learning-induced autonomy of sensorimotor systems. arXiv 1403: 6034. 10.1038/nn.3993 PMC6368853 25849989

[21] Mantzaris AV, Bassett DS, Wymbs NF, Estrada E, Porter MA, et al (2013) Dynamic network centrality summarizes learning in the human brain. Journal of Complex Networks 1: 83–92.

[22] Bassett DS, Porter MA, Wymbs NF, Grafton ST, Carlson JM, et al (2013) Robust detection of dynamic community structure in networks. Chaos 23: 013142. 23556979 10.1063/1.4790830 PMC3618100

[23] Bassett DS, Wymbs NF, Porter MA, Mucha PJ, Grafton ST (2014) Cross-linked structure of network evolution. Chaos 24: 013112. 24697374 10.1063/1.4858457 PMC4108627

[24] Samaria F, Harter A (1994) Parameterisation of a stochastic model for human face identification. 2nd IEEE Workshop on Applications of Computer Vision. Sarasota (Florida).

[25] Martinez A, Benavente R (1998) The AR face database. CVC Technical Report no.24.

[26] Peer P. Computer Vision Laboratory Face Database, University of Ljubljana, Slovenia. URL http://www.lrv.fri.uni-lj.si/facedb.html.

[27] Solina F, Peer P, Batagelj B, Juvan S, Kova J (2003) Color-based face detection in the ‘15 seconds of fame’ art installation. In: Mirage 2003: Conference on Computer Vision/Computer Graphics Collaboration for Model-based Imaging, Rendering, Image Analysis and Graphical Special Effects. pp.38–47.

[28] Minear M, Park D (2004) A lifespan database of adult facial stimuli. Behaviour Research Methodology Instrumentation Computer 36: 630–633. 10.3758/bf03206543 15641408

[29] Weyrauch B, Huang J, Heisele B, Blanz V (2004) Component-based face recognition with 3D morphable models. First IEEE Workshop on Face Processing in Video, Washington, D.C..

[30] Hermundstad AM, Bassett DS, Brown KS, Aminoff EM, Clewett D, et al (2013) Structural foundations of resting-state and task-based neural activity in the human brain. Proceedings of the National Academy of Sciences 110: 6169–6174. 10.1073/pnas.1219562110 PMC3625268 23530246

[31] Aminoff EM, Clewett D, Freeman S, Frithsen A, Tipper C, et al (2012) Individual differences in shifting decision criterion: A recognition memory study. Memory & Cognition 40: 1016–1030. 22555888 10.3758/s13421-012-0204-6

[32] Daducci A, Gerhard S, Griffa A, Lemkaddem A, Cammoun L, et al (2012) The connectome mapper: an open-source processing pipeline to map connectomes with MRI. PLoS ONE 7: e48121. 23272041 10.1371/journal.pone.0048121 PMC3525592

[33] Hagmann P, Cammoun L, Gigandet X, Meuli R, Honey CJ, et al (2008) Mapping the structural core of human cerebral cortex. PLoS Biology 6: e159. 18597554 10.1371/journal.pbio.0060159 PMC2443193

[34] Smith SM, Jenkinson M, Woolrich MW, Beckmann CF, Behrens TE, et al (2004) Advances in functional and structural MR image analysis and implementation as FSL. NeuroImage 23: S208–S219. 15501092 10.1016/j.neuroimage.2004.07.051

[35] Woolrich MW, Jbabdi S, Patenaude B, Chappell M, Makni S, et al (2009) Bayesian analysis of neuroimaging data in FSL. NeuroImage 45: S173–S186. 19059349 10.1016/j.neuroimage.2008.10.055

[36] Jenkinson M, Beckmann CF, Behrens TE, Woolrich MW, Smith SM (2012) FSL. NeuroImage 62: 782–790. 21979382 10.1016/j.neuroimage.2011.09.015

[37] Cox RW (1996) AFNI: software for analysis and visualization of functional magnetic resonance neuroimages. Computers and Biomedical Research 29: 162–173. 8812068 10.1006/cbmr.1996.0014

[38] Jenkinson M, Bannister P, Brady M, Smith S (2002) Improved optimization for the robust and accurate linear registration and motion correction of brain images. NeuroImage 17: 825–841. 12377157 10.1016/s1053-8119(02)91132-8

[39] Power JD, Barnes KA, Snyder AZ, Schlaggar BL, Petersen SE (2012) Spurious but systematic correlations in functional connectivity MRI networks arise from subject motion. NeuroImage 59: 2142–2154. 22019881 10.1016/j.neuroimage.2011.10.018 PMC3254728

[40] Jo HJ, Gotts SJ, Reynolds RC, Bandettini PA, Martin A, et al.. (2013) Effective preprocessing procedures virtually eliminate distance-dependent motion artifacts in resting state fMRI. Journal of Applied Mathematics. 10.1155/2013/935154 PMC3886863 24415902

[41] Greve DN, Fischl B (2009) Accurate and robust brain image alignment using boundary-based registration. NeuroImage 48: 63–72. 19573611 10.1016/j.neuroimage.2009.06.060 PMC2733527

[42] Zalesky A, Fornito A, Harding IH, Cocchi L, Yücel M, et al (2010) Whole-brain anatomical networks: does the choice of nodes matter? NeuroImage 50: 970–983. 20035887 10.1016/j.neuroimage.2009.12.027

[43] Lynall ME, Bassett DS, Kerwin R, McKenna P, Muller U, et al (2010) Functional connectivity and brain networks in schizophrenia. The Journal of Neuroscience 30: 9477–87. 20631176 10.1523/JNEUROSCI.0333-10.2010 PMC2914251

[44] Bassett DS, Nelson BG, Mueller BA, Camchong J, Lim KO (2012) Altered resting state complexity in schizophrenia. NeuroImage 59: 2196–2207. 22008374 10.1016/j.neuroimage.2011.10.002 PMC3254701

[45] Sun FT, Miller LM, D′Esposito M (2004) Measuring interregional functional connectivity using coherence and partial coherence analyses of fMRI data. NeuroImage 21: 647–658. 14980567 10.1016/j.neuroimage.2003.09.056

[46] Cadzow JA (1973) Discrete-Time Systems: An Introduction with Interdisciplinary Applications. Prentice-Hall Englewood Cliffs, NJ.

[47] Fornito A, Zalesky A, Bullmore ET (2010) Network scaling effects in graph analytic studies of human resting-state fMRI data. Frontiers in Systems Neuroscience 4: 22. 20592949 10.3389/fnsys.2010.00022 PMC2893703

[48] Genovese CR, Lazar NA, Nichols TE (2002) Thresholding of statistical maps in functional neuroimaging using the false discovery rate. NeuroImage 15: 870–878. 11906227 10.1006/nimg.2001.1037

[49] Evans T, Lambiotte R (2009) Line graphs, link partitions, and overlapping communities. Physical Review E 80: 016105. 10.1103/PhysRevE.80.016105 19658772

[50] Ahn YY, Bagrow JP, Lehmann S (2010) Link communities reveal multiscale complexity in networks. Nature 466: 761–764. 20562860 10.1038/nature09182

[51] Rosvall M, Esquivel AV, Lancichinetti A, West JD, Lambiotte R (2014) Memory in network flows and its effects on spreading dynamics and community detection. Nature Communications 5: 4630. 10.1038/ncomms5630 25109694

[52] Eagle N, Pentland AS, Lazer D (2009) Inferring friendship network structure by using mobile phone data. Proceedings of the National Academy of Sciences 106: 15274–15278. 10.1073/pnas.0900282106 PMC2741241 19706491

[53] Hochberg Y (1988) A sharper Bonferroni procedure for multiple tests of significance. Biometrika 75: 800–802.

[54] Sporns O, Zwi JD (2004) The small world of the cerebral cortex. Neuroinformatics 2: 145–162. 15319512 10.1385/NI:2:2:145

[55] van den Heuvel MP, Stam CJ, Kahn RS, Pol HEH (2009) Efficiency of functional brain networks and intellectual performance. The Journal of Neuroscience 29: 7619–7624. 19515930 10.1523/JNEUROSCI.1443-09.2009 PMC6665421

[56] Hermundstad AM, Brown KS, Bassett DS, Aminoff EM, Frithsen A, et al (2014) Structurally-constrained relationships between cognitive states in the human brain. PLoS Computational Biology 10: e1003591. 24830758 10.1371/journal.pcbi.1003591 PMC4022461

[57] Bassett DS, Greenfield DL, Meyer-Lindenberg A, Weinberger DR, Moore S, et al (2010) Efficient physical embedding of topologically complex information processing networks in brains and computer circuits. PLoS Computational Biology 6: e1000748. 20421990 10.1371/journal.pcbi.1000748 PMC2858671

[58] Long XY, Zuo XN, Kiviniemi V, Yang Y, Zou QH, et al (2008) Default mode network as revealed with multiple methods for resting-state functional MRI analysis. Journal of Neuroscience Methods 171: 349–355. 18486233 10.1016/j.jneumeth.2008.03.021

[59] Albert NB, Robertson EM, Mehta P, Miall RC (2009) Resting state networks and memory consolidation. Communicative & Integrative Biology 2: 530–532. 20195459 10.4161/cib.2.6.9612 PMC2829828

[60] Dastjerdi M, Foster BL, Nasrullah S, Rauschecker AM, Dougherty RF, et al (2011) Differential electrophysiological response during rest, self-referential, and non-self-referential tasks in human posteromedial cortex. Proceedings of the National Academy of Sciences 108: 3023–3028. 10.1073/pnas.1017098108 PMC3041085 21282630

[61] Fransson P, Marrelec G (2008) The precuneus/posterior cingulate cortex plays a pivotal role in the default mode network: Evidence from a partial correlation network analysis. NeuroImage 42: 1178–1184. 18598773 10.1016/j.neuroimage.2008.05.059

[62] Cauda F, Geminiani G, D′Agata F, Sacco K, Duca S, et al (2010) Functional connectivity of the posteromedial cortex. PLoS ONE 5: e13107. 20927345 10.1371/journal.pone.0013107 PMC2948030

[63] Corbetta M, Shulman GL (2002) Control of goal-directed and stimulus-driven attention in the brain. Nature Reviews Neuroscience 3: 201–215. 11994752 10.1038/nrn755

[64] Fox MD, Corbetta M, Snyder AZ, Vincent JL, Raichle ME (2006) Spontaneous neuronal activity distinguishes human dorsal and ventral attention systems. Proceedings of the National Academy of Sciences 103: 10046–10051. 10.1073/pnas.0604187103 PMC1480402 16788060

[65] Seeley WW, Menon V, Schatzberg AF, Keller J, Glover GH, et al (2007) Dissociable intrinsic connectivity networks for salience processing and executive control. The Journal of Neuroscience 27: 2349–2356. 17329432 10.1523/JNEUROSCI.5587-06.2007 PMC2680293

[66] Nobre AC, Sebestyen GN, Gitelman DR, Mesulam MM, Frackowiak RS, et al (1997) Functional localization of the system for visuospatial attention using positron emission tomography. Brain 120: 515–533. 9126062 10.1093/brain/120.3.515

[67] Corbetta M, Miezin FM, Shulman GL, Petersen SE (1993) A PET study of visuospatial attention. The Journal of Neuroscience 13: 1202–1226. 8441008 10.1523/JNEUROSCI.13-03-01202.1993 PMC6576604

[68] Hopfinger JB, Buonocore MH, Mangun GR (2000) The neural mechanisms of top-down attentional control. Nature Neuroscience 3: 284–291. 10700262 10.1038/72999

[69] Shomstein S, Behrmann M (2005) Goal-directed attentional orienting in patients with dorsal parietal lesions. Journal of Vision 5: 690–690.16356079

[70] Levy I, Hasson U, Avidan G, Hendler T, Malach R (2001) Center-periphery organization of human object areas. Nature Neuroscience 4: 533–539. 11319563 10.1038/87490

[71] Nestor A, Behrmann M, Plaut DC (2013) The neural basis of visual word form processing: a multivariate investigation. Cerebral Cortex 23: 1673–1684. 22693338 10.1093/cercor/bhs158 PMC13377679

[72] Turkeltaub PE, Eden GF, Jones KM, Zeffiro TA (2002) Meta-analysis of the functional neuroanatomy of single-word reading: method and validation. NeuroImage 16: 765–780. 12169260 10.1006/nimg.2002.1131

[73] Polk TA, Farah MJ (2002) Functional MRI evidence for an abstract, not perceptual, word-form area. Journal of Experimental Psychology 131: 65–72. 11900104 10.1037//0096-3445.131.1.65

[74] Rauschecker AM, Bowen RF, Perry LM, Kevan AM, Dougherty RF, et al (2011) Visual feature-tolerance in the reading network. Neuron 71: 941–953. 21903085 10.1016/j.neuron.2011.06.036 PMC3180962

[75] Vogel AC, Miezin FM, Petersen SE, Schlaggar BL (2011) The putative visual word form area is functionally connected to the dorsal attention network. Cerebral Cortex: bhr100. 10.1093/cercor/bhr100 PMC3278314 21690259

[76] Haxby JV, Hoffman EA, Gobbini MI (2002) Human neural systems for face recognition and social communication. Biological Psychiatry 51: 59–67. 11801231 10.1016/s0006-3223(01)01330-0

[77] Kanwisher N, McDermott J, Chun MM (1997) The fusiform face area: a module in human extrastriate cortex specialized for face perception. The Journal of Neuroscience 17: 4302–4311. 9151747 10.1523/JNEUROSCI.17-11-04302.1997 PMC6573547

[78] Gauthier I, Tarr MJ, Moylan J, Skudlarski P, Gore JC, et al (2000) The fusiform “face area” is part of a network that processes faces at the individual level. Journal of Cognitive Neuroscience 12: 495–504. 10931774 10.1162/089892900562165

[79] McCarthy G, Puce A, Gore JC, Allison T (1997) Face-specific processing in the human fusiform gyrus. Journal of Cognitive Neuroscience 9: 605–610. 23965119 10.1162/jocn.1997.9.5.605

[80] Saygin ZM, Osher DE, Koldewyn K, Reynolds G, Gabrieli JDE, et al (2012) Anatomical connectivity patterns predict face selectivity in the fusiform gyrus. Nature Neuroscience 15: 321–327. 22197830 10.1038/nn.3001 PMC3267901

[81] Cole MW, Yarkoni T, Repovš G, Anticevic A, Braver TS (2012) Global connectivity of prefrontal cortex predicts cognitive control and intelligence. The Journal of Neuroscience 32: 8988–8999. 22745498 10.1523/JNEUROSCI.0536-12.2012 PMC3392686

[82] Schneidman E, Berry MJ, Segev R, Bialek W (2006) Weak pairwise correlations imply strongly correlated network states in a neural population. Nature 440: 1007–1012. 16625187 10.1038/nature04701 PMC1785327

[83] Zalesky A, Fornito A, Bullmore E (2012) On the use of correlation as a measure of network connectivity. NeuroImage 60: 2096–2106. 22343126 10.1016/j.neuroimage.2012.02.001

