# Supplementary material for: Brain Network Adaptability across Task States
Source: PLoS Comput Biol. 2015 Jan 8;11(1):e1004029. doi: 10.1371/journal.pcbi.1004029 (PMC4287347; doi:10.1371/journal.pcbi.1004029)
Supplement: S1 Text — Supplementary methodological information. Discussion of the effects of time window selection and brain region size on the results, with accompanying figures. (PDF) [file pcbi.1004029.s001.pdf]

Text S1: Supplementary Methodological Information

In this supplementary document, we include the following material to support the work described in the main text.

1. An examination of hyperedge properties across analyses that vary either the length or number of samples in time windows used for dynamic network construction.

2. Figure 1: Comparison of hyperedge size distributions in analyses with differing time window selection procedures.

3. A discussion of hyperedge node degree and its relation to brain region size.

4. Figure 2: Relation of hyperedge node degree to brain region size.

Examination of time window selection across different TRs

fMRI data was sampled during rest and attention tasks with a TR of 2 seconds, and during memory tasks with a TR of 2.5 seconds. As a result, when choosing time windows for our dynamic network, we could not hold both the time window length and the number of samples per window constant over the entire experiment. To ensure that a discontinuity in either window length or window samples does not drive our results, we performed two separate analyses: one with a constant time window length of 60 seconds but a change in the number of samples per window, and the other with a constant number of samples in all windows but a change in the window length. The hyperedge size distributions from both analyses are shown in Fig. 1.

The distribution with the number of samples per time window held constant (Fig. 1B) has slightly smaller hyperedges in both the "small" and "large" regimes than the distribution with constant window lengths (Fig. 1A), likely driven by the longer time windows (75 rather than 60 seconds) in the memory tasks. Despite these small differences between the distributions, our results are quite robust to variation of the window length selection strategy, showing a very similar distribution shape and overall number of hyperedges in both. We choose to use results from the analysis with constant time window lengths in the main text of the paper, so that each time layer in our dynamic network covers the same duration of time.

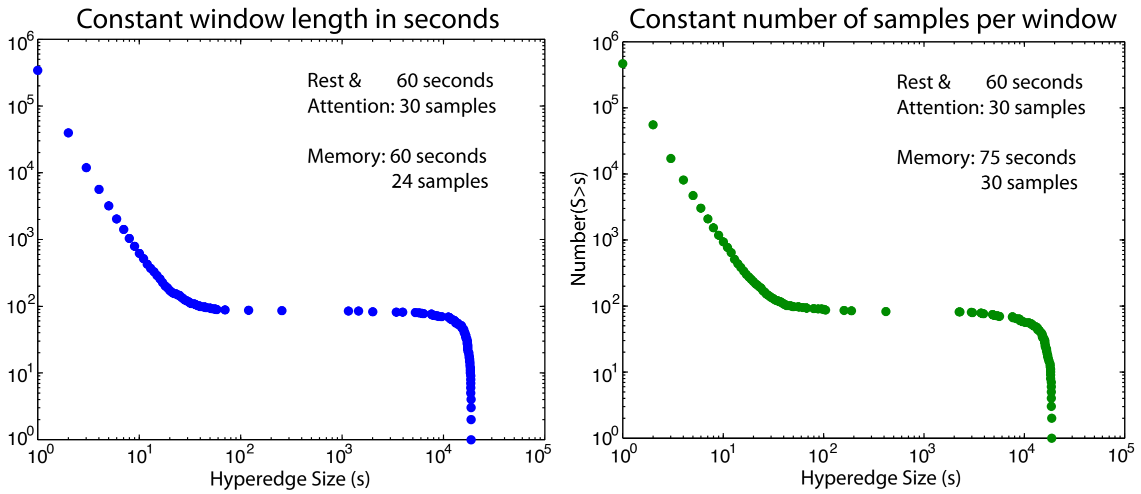

Figure 1: **Cumulative Hyperedge Size Distributions: Left:** Cumulative hyperedge size distribution as presented in the main text, from an analysis in which time windows were held at a constant length of 60 seconds across tasks with different TRs. **Right:** Cumulative hyperedge size distribution from an analysis in which the number of data points used to compute the correlation in each time window was held constant at 30 samples across tasks. In this case, time windows in memory tasks covered 75 seconds. These two analyses produce very similar results, with slightly fewer large hyperedges and more small hyperedges in the right-hand distribution.

Correlation of hyperedge node degree with brain region size

The hyperedge node degree is a measure of the number of hyperedges in which a node participates. We expect it to contain information about a given node's likelihood of co-evolving with other nodes in the network. To check whether a node's hyperedge degree is influenced by the size of that node, we investigated the correlation between physical node size and hyperedge node degree (presented in Fig. 2).

We find that the correlation between size and hyperedge node degree is not extremely strong (Spearman's $\rho = 0.401$, Pearson's $r = 0.324$). However, the p-values for these relationships are highly statistically significant (Spearman p-value $= 7.01 \times 10^{-9}$, Pearson p-value $= 4.12 \times 10^{-6}$).

While the hyperedge node degree is somewhat influenced by node size, the relatively weak correspondence shown in Fig. 2 indicates that the hyperedge degree is not simply a reflection of node size alone; we also expect it to include information on the extent of the node's co-evolution with other brain regions. We have explicitly constructed a "hybrid atlas, as described in detail in the main text, in order to minimize the variation in brain region size across both subjects and regions, and control the effect of variations in node size on our results as much as possible.

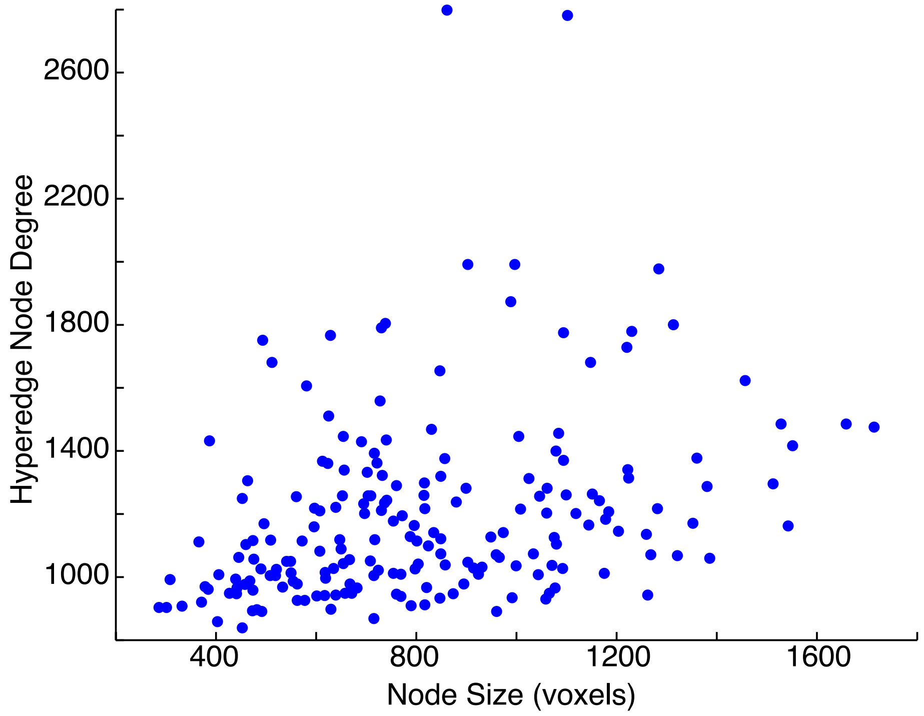

Figure 2: **Relation of hyperedge node degree to brain region size:** A scatter plot of the size of each node (brain region) in voxels plotted against its hyperedge node degree. Each voxel is a cubic volume with sides of 2mm. The two are not especially strongly correlated (Spearman's $\rho = 0.401$, Pearson's $r = 0.324$), but the p-values for these relationships are highly significant (Spearman p-value $= 7.01 \times 10^{-9}$, Pearson p-value $= 4.12 \times 10^{-6}$).
